# 3-D health trajectories and related childhood predictors among older adults in China

**DOI:** 10.1038/s41598-021-89354-6

**Published:** 2021-05-10

**Authors:** Chaoping Pan, Cen Wang, Bhawana Shrestha, Peigang Wang

**Affiliations:** School of Health Sciences, Wuhan University, 115 Donghu Road, Wuhan City, 430071 Hubei Province China

**Keywords:** Diseases, Risk factors

## Abstract

This study aimed to identify the multi-trajectories of 3-D health of older adults in China and to explore whether the childhood predictors are associated with 3-D health trajectory. Data came from five waves of the China Health and Retirement Longitudinal Study (CHARLS, 2011 to 2018). A multi-trajectory modeling approach was carried out to jointly estimate the trajectories of 3-D health. A multinomial regression model was used to investigate the relationships between childhood predictors and the joint trajectories. We identified three typical joint 3-D health trajectories. Female, childhood health, maternal and paternal educations, childhood friendships, family and neighborhood predictors could all affect 3-D health trajectories of older adults directly or indirectly through adult variables. The 3-D health trajectories showed increasing trends, thus the government should perform more interventions toward the childhood predictors for better health of older adults.

## Introduction

Facing rapid aging in China, nearly 40 million and 180 million older adults had a disability and chronic disease in 2019; and nearly 22.7% had depression^1^. Preventing disability, chronic disease and depression can be a great benefit for the improvement of health and life quality of old adults, and reducing the huge burden for the national support system^1–3^. Older adults may own different health trajectories: Some may maintain good health, while others may become ill at an early age. Interventions that do not consider the various trajectories of illnesses among older adults could be ineffective.

As chronic and non-communicable diseases become the dominant diseases in China, early predictors will play more critical roles in the health of older adults^4,5^. The interventions of early health determinants can promote the health of old adults and largely reduce the old-age care burden^6^. The Chinese government has issued many policies to promote health aging such as the “Actively Responding to Aging” strategy and the “Healthy China 2030” plan. “Actively Responding to Aging” strategy means to prevent disease rather than treating it after illness, and the “Healthy China 2030” plan puts forward the concept of “life-cycle health management”, and those policies strengthen the critical role of the early interventions in promoting the health of older adults. However, the precise implementation of interventions requires understanding which early factors will affect the health of older adults.

In this paper, we proposed a 3-D health trajectory model, which assumes that the trajectories of disability, disease and depression are related to each other. And by using the 3-D health trajectory model, we tried to better explore the health trajectories of older adults. The paper also explored whether the early predictors such as childhood friendship, family, and neighborhood predictors would influence the later 3-D health trajectories and the indirect effects of those earlier variables on the 3-D health trajectories through adult variables.

### Literature review

As the multidimensional nature of health and aging^7^, recently more researches have paid attention to the relationship between diseases, such as chronic diseases, disability, and depression, and indicated that estimating one direction of the interrelationship between diseases may be misleading^8–10^. Several studies have explored disease, disability or depression trajectories among older adults, but they were estimated separately as if three independent processes^7,11^. To our knowledge, none of the studies examined the disability, depression and disease trajectories jointly and how the three components evolved over time.

Though the change of the human disease spectrum makes early social determinants more critical for later human health, studies focusing on earlier social predictors of health are still not enough^12^. Several researches have explored the predictors of health trajectories of older adults by considering the predicators at baseline^13,14^ and a few included childhood variables, such as childhood health or childhood socioeconomic status, but they did not focus on the childhood predicators^15^. Previous studies also showed that those who have more advantages in childhood will also be healthier in later life than those who do not have the advantages, which means that childhood risk factors may affect the later health through adult variables^16–18^. While previous studies showed that childhood friendship, family and neighborhood predictors played critical roles in health^17,19–21^, we are unaware of any studies that focused on the direct or indirect effects through adult variables of those variables on 3-D health trajectories of older adults.

### Theory and research questions

Many theories emphasized the effects of early factors on health, such as cumulative disadvantage theory, stress process theory and the life course theory^22^. Integrating elements of those theories, the cumulative inequality theory is a core theory to explain the influence of early factors on the health of later life. The theory specifically focuses on early health risk factors from the perspective of life course, and suggests that early factors may not only parlay their advantage into further advantage through the accumulation of resources, but also are associated with crucial socialization processes, including the development of self-esteem, role-modeling of vocational and marital behavior, the internalization of values and norms, which can finally affect the health of later life by influencing continued exposure to stressors and behaviors unconducive to later health (e.g., drinking)^23,24^.

Though many theories emphasized the importance of early factors to the health of later life, few researches combined multi-trajectory modeling approach with the childhood predictors of later health, so the effects of the childhood predictors on the joint health trajectories of older adults are still not clear. In this paper, two main research questions are formulated to advance our understanding of how early factors influence the 3-D health trajectories of older adults: the first, what are characteristics of joint trajectories of 3-D health in older adults? As facing an increasingly aging population in China, we anticipate that the trajectories of 3-D health have increasing trends. The second, how do childhood predictors affect the health of older adults? We assume that childhood friendship, family and neighborhood predictors can affect the 3-D health trajectories of older adults directly or indirectly through adult variables.

## Results

### Health trajectories of older adults

Table 1 shows the results from the best-fitting base model, which was divided into three groups. In the 3-D health model, time was modeled with quadratic specifications for all three disability and depression groups, linear specifications for all three disease groups. The joint model shared the same grouping samples and maximum likelihood estimation assigned 42.57%, 38.07%, and 19.36% group memberships for the low, middle and high 3-D health group, respectively.

**Table 1:** Maximum likelihood estimates of disability, disease and depression from the best base model.

Parameter	The low 3-D health group	The middle 3-D health group	The high 3-D health group
Disability			
Intercept	− 1.567 (0.062)***	− 0.755 (0.099)***	1.313 (0.022)***
Linear scaled time	− 0.055 (0.034)	0.069 (0.024)**	− 0.009 (0.012)
Quadratic scaled time	0.033 (0.004)***	− 0.013 (0.003)***	0.007 (0.002)**
Chronic disease			
Intercept	0.619 (0.041)***	1.645 (0.046)***	2.326 (0.060)***
Linear scaled time	0.207 (0.009)***	0.236 (0.010)***	0.287 (0.014)***
Depression			
Intercept	1.479 (0.016)***	2.367 (0.012)***	2.687 (0.011)***
Linear scaled time	− 0.031 (0.009)***	− 0.043 (0.006)***	− 0.029 (0.007)***
Quadratic scaled time	0.005 (0.001)***	0.008 (0.001)***	0.006 (0.001)***
Group membership	42.57	38.07	19.36
BIC	− 99,839.10		

Standard errors are in parentheses.

*p < 0.05; **p < 0.01; ***p < 0.001.

Table 2 shows the model fit of the best base model. The final average posterior probabilities were 0.96 for the low and high groups, 0.94 for the middle group and all well above the 0.7 criterion; the odds of correct classification were 17.79, 9.63 and 5.76, all above the 5 criterion; the differences between estimated group probabilities and the actual proportion using the maximum probability rule, were almost identical for each group.

**Table 2:** The model fit of the best base model.

Trajectory groups	AvePP	OCC	|π–P|
Low	0.96	17.79	< 0.01
Middle	0.94	9.63	< 0.01
High	0.96	5.76	< 0.01

AvePP, average posterior probability; OCC, odds of correct classification; P, actual proportion of subjects assigned to each trajectory group using the maximum probability rule; π, posterior probability of group membership.

**Figure 1:**
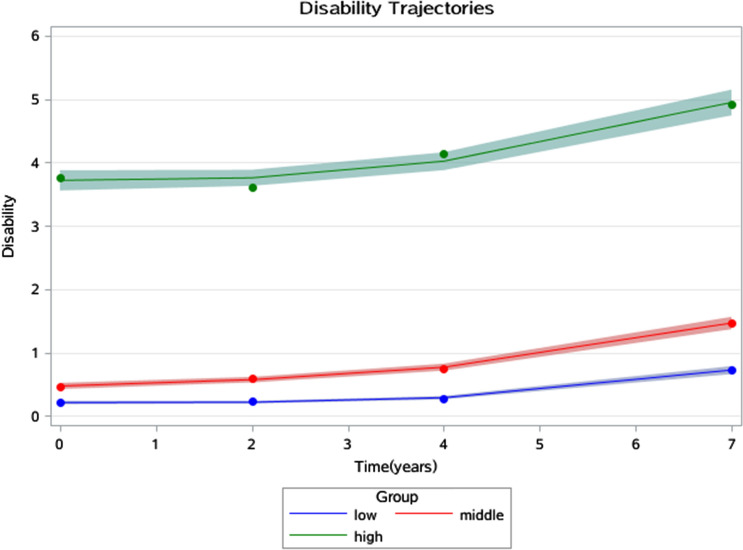
Disability trajectories among a sample of older individuals that survived a 7-year period in China.

**Figure 2:**
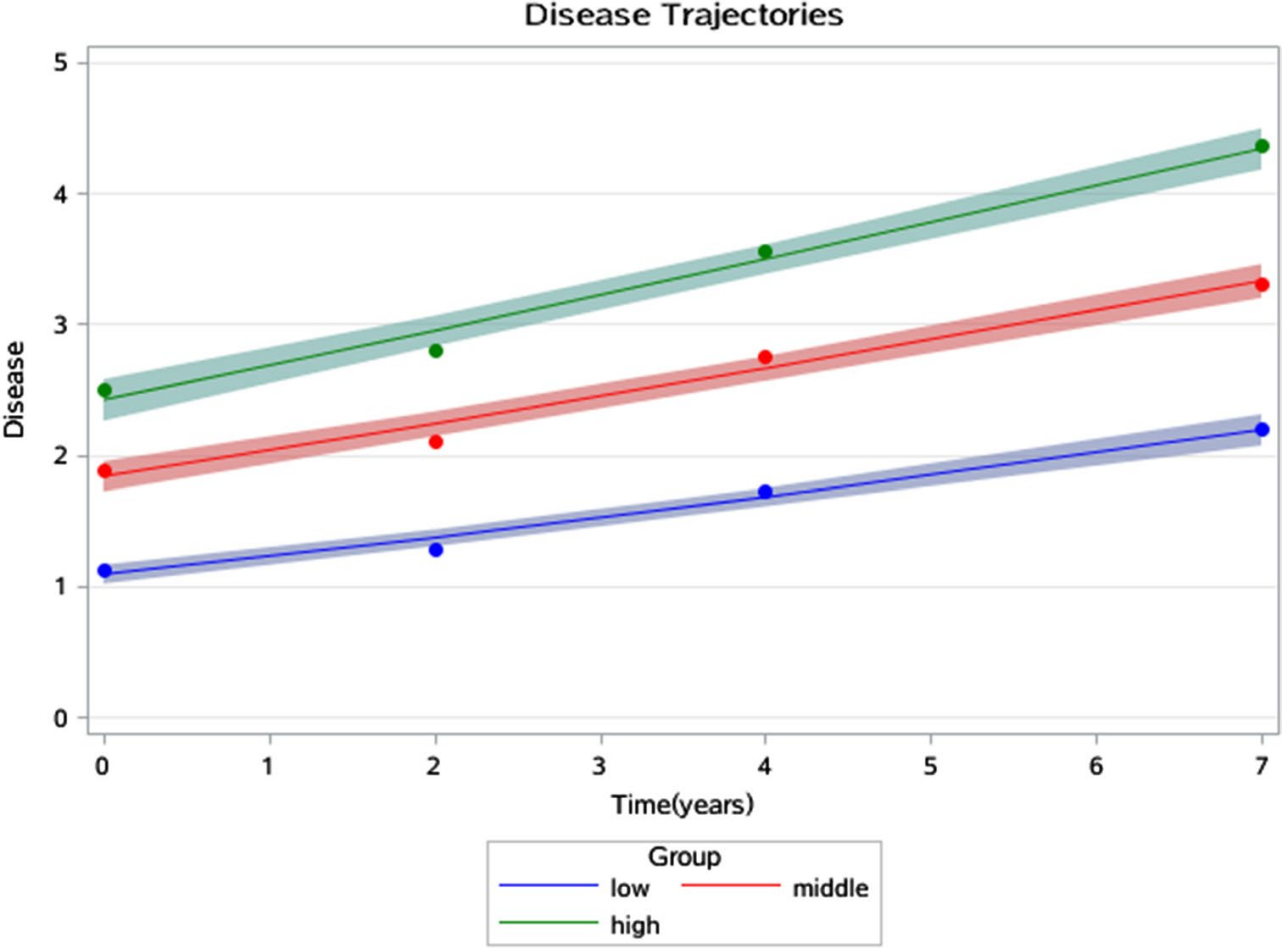
Disease trajectories among a sample of older individuals that survived a 7-year period in China.

**Figure 3:**
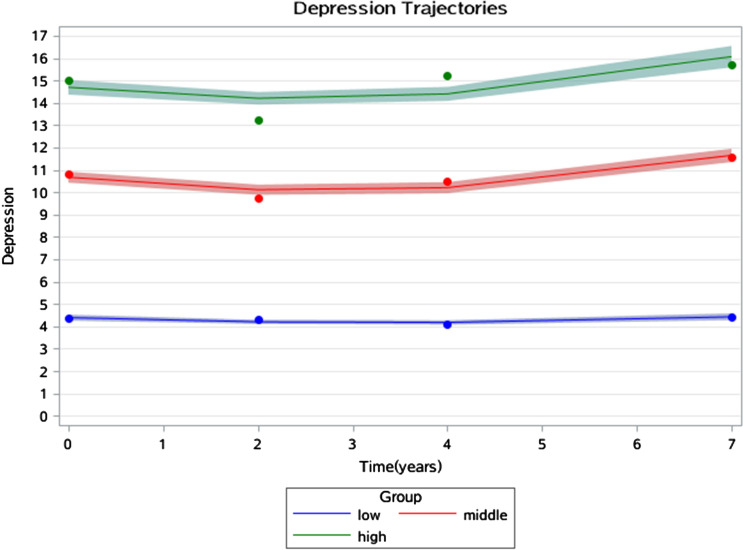
Depression trajectories among a sample of older individuals that survived a 7-year period in China.

Figures 1, 2 and 3 show the joint trajectories of 3-D health. The average disability trajectories were 0.21, 0.47, and 3.72 points at the baseline which finally risen to 0.72, 1.46, and 4.95 points in 7 years. These corresponded with 1.09, 1.84 and 2.42 points of disease trajectories at the baseline which then developed to 2.19, 3.33, and 4.34 points in 7 years, and corresponded with 4.39, 10.67 and 14.69 points of depression trajectories at baseline which then developed to 4.43, 11.65 and 16.06 points in 7 years.

### Childhood characteristics of older adults on 3-D health trajectory groups

Table 3 shows all childhood variables that significantly differed among the three trajectory groups. One-way ANOVA or chi-squared test was used to examine the significances of childhood variables among trajectories. The results show that older male adults had a higher probability to be in the low 3-D health group. Older adults with better family economic status during childhood had the same tendency to follow the lower 3-D health group. Older adults whose mother and father had higher education levels were more likely to be on the low 3-D health group. Older adults who had better neighborhood safety, a more helpful neighborhood, better neighborhood cleanliness, better health, lower loneliness, playing more with friends, and bullied less by kids during childhood had a higher probability of following the low 3-D health group.

**Table 3:** Childhood characteristics of older adults on three disability trajectory groups.

Variables	N	Group 1	Group 2	Group 3	*Χ*^2^/*F*	*p* value
Female	4310	695 (37.96)	850 (52.15)	533 (62.78)	159.39	< 0.001
Childhood health	4310	2.51 (0.96)	2.76 (0.98)	2.80 (0.99)	38.95	< 0.001
Maternal education	4310	1.16 (0.69)	1.09 (0.48)	1.04 (0.29)	16.35	< 0.001
Paternal education	4310	1.99 (1.50)	1.80 (1.34)	1.70 (1.33)	15.22	< 0.001
Family economic status	4310	3.39 (0.93)	3.56 (0.99)	3.70 (1.01)	33.24	< 0.001
Parent relationship	4310	2.46 (1.17)	2.67 (1.23)	2.70 (1.26)	17.04	< 0.001
Neighborhood safety	4310	1.54 (0.67)	1.67 (0.77)	1.70 (0.80)	21.66	< 0.001
Neighborhood helping	4310	1.65 (0.76)	1.81 (0.86)	1.92 (0.94)	33.34	< 0.001
Neighborhood cleanliness	4310	2.23 (0.74)	2.30 (0.79)	0.37 (0.85)	9.03	< 0.001
Loneliness	4310	3.73 (0.72)	3.52 (0.93)	3.40 (1.03)	49.88	< 0.001
Playing with friends	4310	1.74 (1.11)	2.03 (1.23)	2.21 (1.27)	53.80	< 0.001
Bullied by kids	4310	3.74 (0.63)	3.61 (0.81)	3.56 (0.86)	53	< 0.001

Continuous variables are shown as mean (SD) and categorical variables are shown as frequency (%).

### Childhood predictors and 3-D health trajectories

Table 4 shows the association of childhood demographic variables, family, neighborhood, and friendship predictors with joint 3-D health trajectory group memberships. Model 1 included all childhood variables. The results showed that being female made older adults 88.8% and 199.6% more likely to be in the middle and high 3-D health groups compared with the low 3-D health group. For every point of children’s health score reduction, the probabilities of being in the middle and high 3-D health groups were 22.3% and 24.1% higher than that of the low 3-D health group. Every grade increment in maternal education level made older adults 30.4% less probable to be in the high 3-D health group than in the low 3-D health group. Every grade increment in paternal education level made older adults 5.4% and 7.5% less probable to be in the middle and high 3-D health groups than in low 3-D health group. Childhood economic level decreasing every grade made older adults 7.8% and 19.3% more likely to be in the middle and high 3-D health groups than in low 3-D health group. Parent relationship level decreasing every grade made older adults 9.8% and 9.1% more probable to be in the middle and high 3-D health groups compared with the low 3-D health group.

**Table 4:** Effects of early factors on the 3-D health trajectory model among a sample of older adults survived 7 years in China.

Variables	Model 1		Model 2	
	OR		OR	
	Group 2	Group3	Group 2	Group3
	Vs	Vs	Vs	Vs
	Group 1	Group1	Group 1	Group1
Female	1.888***	2.996***	1.959***	2.713***
Childhood health	1.233***	1.241***	1.236***	1.242***
Maternal education	0.896	0.696**	0.948	0.800
Paternal education	0.946*	0.925*	0.975	0.976
Family economic status	1.078*	1.193***	1.045	1.149**
Parent relationship	1.098**	1.091*	1.095**	1.100*
Neighborhood Safety	1.163**	1.131*	1.156**	1.098
Neighborhood Helping	1.075	1.156**	1.073	1.108
Neighborhood cleanliness	1.055	1.168**	1.074	1.200**
Loneliness	0.807***	0.755***	0.836***	0.793***
Playing with friends	1.148***	1.243***	1.102**	1.141***
Bullied by kids	0.828***	0.798***	0.815***	0.767***
Age			1.007	1.047***
Education			0.941**	0.809***
Residence			1.612***	1.533***
Marriage status			0.91	0.854
Smoking			1.073	1.152
Drinking			1.140	1.088
Medical insurance			0.766	0.698
Pension			0.849*	0.722***
BIC	8756.69		8564.09	

*OR* odds ratio, *CI* confidence interval, *BIC* Bayesian Information Criterion. *p < 0.05; **p < 0.01; ***p < 0.001.

The neighborhood safety score during childhood decreasing every point made older adults 16.3% and 13.1% more likely to be in the middle and high 3-D health groups than in the low 3-D health group. Neighborhood helping score during childhood decreasing every point made older adults 15.6% more likely to be in the high 3-D health group than in the low 3-D health group. Neighborhood cleanliness score during childhood decreasing every point made older adults 16.8% more likely to be in the high 3-D health group than in the low 3-D health group.

Every point decrement of loneliness score during childhood made older adults 19.3% and 24.5% less likely to be in the middle and high 3-D health groups than in low 3-D health group. Every point of increment of playing with friends score during childhood older adults made older adults 14.8% and 24.3% less likely to be in the middle and high 3-D health groups than in low 3-D health group. Every point decrement of bullied by kids score during childhood made older adults 17.2% and 20.2% less likely to be in the middle and high 3-D health groups than in low 3-D health group.

To explore the mediate effects of adulthood variables, model 2 included the childhood variables plus adulthood variables. The significances of maternal education, paternal education, family economic status, neighborhood safety, neighborhood helping, and playing with friends were vanished or decreased after added adulthood variables, while the impacts of other variables on group memberships of 3-D health trajectory were changed only a little.

### Sensitive analysis

To assess how 3-D health trajectories might be affected by not jointly modeling attrition due to death, we conducted the model without jointly modeling mortality. The group memberships showed only a few differences with jointly modeling mortality, with low group (42.43%vs 42.57%), middle group (38.16%vs 38.07%) and high group (19.41% vs 19.36%). We also performed the model including mortality and intermittent missing data, and only a few differences in group memberships were observed compared with model of jointly modeling mortality, with low group (43.02%vs 42.57%), middle group (37.39%vs 38.07%) and high group (19.58% vs 19.36%). The joint trajectories nearly had the same trends in three models. The results of sensitivity analysis were in line with empirical research, which indicated a good robustness of our research.

## Discussions

Our first research question asked what characteristics of joint trajectories of 3-D health in older adults were. We identified three joint trajectories of 3-D health according to the model fit index and explanation of the results. The results did not coincide with other studies. Martin et al. found a more rapid increment of disability trajectory^15^ and Liang found four depression trajectories^11^. Our results showed that the 3-D health trajectories had increasing trends and the disease had the most obvious increment over time than disability and depression. To better achieve the objective of active aging, the Chinese government should strengthen the implementation of intervention policies such as “Healthy China Action 2030” toward 3-D health of older adults, especially interventions for chronic diseases.

The second question asked how childhood predictors affected the health of older adults. The results revealed that being female was fragile to have a higher 3-D health trajectory (see model 1 in Table 3), which was coincident with other studies that used depression as the outcome variable^11,14^. But the result was different from the study that used disability as outcome variables, which found a weaker association between sex and disability trajectories^15^. Model 2 in Table 3 showed that the significant level of female was not changed compared with model 1 which means that females are more likely to be associated with joint health trajectory directly. In traditional China, the idea of “male supremacy” has resulted in females having fewer resources than men to maintain childhood health, which indicates that the Chinese government should better protect the health equity of girls such as education equity, especially left-behind, disabled and poor girls.

Worse childhood health was associated with a higher 3-D health trajectory (see model 1 in Table 3), which means that improving childhood health is important to the better 3-D health of old adults. A previous study found childhood health could affect disability trajectories indirectly through adult variables^15^. In this study, we used 3-D health trajectories as dependent variables and found different results. Model 2 in Table 3 showed that the significant level of childhood health was not changed compared with model 1, which means that childhood health is more likely to be associated with 3-D health trajectory membership directly. The inclusion of disease and depression as a dependent variable in our study may strengthen the direct effect of childhood health. Therefore, the government and society should not only focus on the health services for older adults, but also for children.

A previous cross-section study showed maternal education could affect the health of Chinese old adults indirectly through adult variables, while paternal education could not affect the health of Chinese old adults by using CHARLS data^24^. In this study, we used 5 waves of CHARLS data, and found that higher maternal and paternal educations were both associated with lower 3-D health trajectories (see model 1 in Table 3). Model 2 in Table 3 showed that the significant levels of maternal and paternal educations were vanished compared with model 1, which means that maternal and paternal educations are more likely to be associated with group memberships of joint health trajectory indirectly through adult variables. So, the Chinese government can extend compulsory education from junior high school to senior high school, which is not only for wellbeing of one generation, but also for the health of their children.

Previous studies showed the result of the association between childhood family economic status and later health was still inconsistent^5,24^. The study found that better childhood family economic status was associated with better 3-D health (see model 1 in Table 3). When compared with model 2 in Table 3, the significant level of childhood family economic status in model 2 reduced but still significant, which means childhood family economic status can affect the 3-D health trajectory membership both directly and indirectly through adult variables. Based on the result, the government should protect the health equity of children who are from poor economic family, such as equity of nutrition, education and access of health services.

Previous studies found that the childhood parent–child relationship could affect later health^17^. But when we added the childhood parent–child relationship variable to model 1, we did not find the relationship (result not shown). Instead, we found better parent relationship during childhood was related to better 3-D health trajectories of older adults (see model 1 in Table 3), and when added the adult variables to model 2 in Table 3, the significant levels of the OR values were not changed, which means that parent relationship during childhood is more likely to be associated with group memberships of 3-D health trajectory directly. A harmonious family atmosphere during childhood should be created for better health of later life.

Better safety of neighborhood was associated with better 3-D health trajectories of older adults (see model 1 in Table 3), and when added the adult variables to model 2 in Table 3, the significant level of the OR values vanished on high group versus low group and the significant level of the OR values was not changed on middle group versus low group compared with model 1, which means that safety of neighborhood can be associated with 3-D health trajectory membership both directly and indirectly through adult variables. So, it is necessary to strengthen protections of public security in neighborhood where children grow up.

More neighborhood helping during childhood was associated with better 3-D health trajectories of older adults (see model 1 in Table 3), and when added the adult variables to model 2 in Table 3, the significant level of the OR values was vanished, which means that childhood neighbor helping is more likely to affect group membership of joint health trajectory indirectly through adult variables. So the government should advocate for neighborhoods helping for better health of older adults.

Better cleanliness of childhood neighborhood was associated with better 3-D health of older adults (see model 1 in Table 3), and when added the adult variables to model 2 in Table 3, the significant level of the OR values was not changed compared with model 1, which means that cleanliness of childhood neighborhood is more likely to be associated with joint health trajectory membership directly. The Chinese ministry of health should strengthen implementation of policies such as “healthy city” and “new rural” policies to improve the cleanliness of neighborhoods.

Every level decrement of childhood loneliness and bullied by kids scores and every level increment of playing with friends score were all associated with the better 3-D health trajectories of older adults (see model 1 in Table 3), and when added the adult variables to model 2 in Table 3, the significant levels of the OR values showed nearly no change compared with model 1, which means that childhood loneliness, playing with friends, bullied by kids are more likely to affect the memberships of joint health trajectory group directly. The results indicate the importance of friendships and mental health during childhood to health of older adults. As children spend most of the time living in school and family, it is critical to strengthen the punishments of school bullying. Family and society should also pay attention to mental health of children.

### Limitations

Several important limitations persistently haunting the field of trajectory modeling and disability researches were still unavoidable. First, we did not include the time-varying indicators when we estimated the multi-trajectory, which might alter our findings. So, further studies are needed.

Second, the variables we used were from self-reported surveys, which may cause bias. However self-reported data (e.g., disease, depression, disability) is commonly used in health trajectory researches of older adults and it can reflect personal status interacting with the real world more accurately^25^.

Third, it may lose some information as we used the summative scores to represent the disability, disease and depression. However, previous studies widely used the summative scores in the health researches of older adults and proved the results of summative scores are acceptable and consistent^8,9,26,27^.

## Conclusions

This study contributes to the existing research in three respects: first, a large five-wave national representative panel data is used and allows for a better investigation of 3-D health trajectories and its childhood predictors of older adults.

Second, it is the first paper to carry out the joint trajectory model to explore the trajectory of 3-D health of older adults and find an increasing trajectory of joint health trajectory. The results are meaningful for understanding the course of 3-D health of older adults.

Third, it is the first paper focusing on the childhood predictors of the health trajectory of older adults. The paper adds contributions to the cumulative inequality theory of how childhood predictors affect the joint 3-D health trajectories of older adults.

Fourth, the paper can provide a new perspective for the Chinese ministry of health to precisely issue intervention policies for “Actively Responding to Aging” and “life-cycle health management” strategies according to the early factors of 3-D health and finally improve wellbeing of older adults.

## Materials and methods

### Study population and measurements

CHARLS is a longitudinal survey that aims to be representative of the residents in mainland China aged 45 and older, with no upper age limit. The national baseline survey was conducted in 2011–12, with wave 2 in 2013, wave 3 in 2014, wave 4 in 2015 and wave 5 in 2018. Wave 4 was a life history survey. To ensure sample representativeness, the CHARLS baseline survey covered 150 countries/districts, 450 villages/urban communities, across the country, involving 17,708 individuals in 10,257 households, reflecting the mid-aged and older Chinese population collectively. More details of CHARLS were described in the study of Ferraro et al.^28^. In this study, the data of 2011, 2013, 2014, 2015 and 2018 waves were used and the individuals older than 60 were included. After excluded the missing data and the individuals younger than 60, the number of each wave was: wave 2011, n = 4310; wave 2013, n = 4106; wave 2015, n = 4046; wave 2018, n = 3588. Since wave 2014 is a life history survey, the participants were the same as wave 2011.

Attrition is a particular concern in analyzing longitudinal data. In this paper, intermittent missing data was assumed as missing at random (MAR) and maximum likelihood estimates was applied to handle it. A total of 51% attrition by the end of the survey was caused by mortality. Considering the attrition due to death may affect the results, we used a multi-trajectory model to model mortality as a joint part of the disability trajectories^29^, which allowed calculating trajectory-specific attrition rates due to death and adjusting the trajectory group membership probabilities.

### Dependent variables

The degree of disability was measured by ADL and IADL. ADL was measured by a 6-item summary, which includes bathing, dressing, eating, getting in/out of bed, using the toilet, and controlling urination. IADL included using the phone, managing money, taking medications, shopping for groceries and preparing hot meals. Disability was dichotomized into no difficulty (0) and some difficulty (1) in performing the tasks. As recommended by previous studies^26,27^, we combined ADL and IADL disabilities in a simple sum to enhance the sensitivity of scaling. So the total score of the severity of disability was 0 to 22.

Depression was measured by the CES-D-10 scale and composed of ten questions including “feeling depressed”, “feeling that everything was an effort”, “whether the respondent’s sleep was restless”, “feeling lonely” and so on^9^. The code was from never (0) to all of the time (3). So the total score of depression ranged from 0 to 30, with higher scores indicating the respondent felt more depressed.

According to other studies^8^, disease was assessed by a sum of 13 different chronic diseases. Each chronic disease was reported by asking “Have you been diagnosed with the following conditions by a doctor”, these chronic diseases were: hypertension, diabetes, cancer, chronic lung disease, heart disease, stroke, emotional problem, arthritis, dyslipidemia, liver disease, kidney disease, digestive disease, and asthma. The answer to each question was coded “no” (0) or “yes” (1). So the total score of disease ranged from 0 to 13.

### Independent variables

Sex was coded as “male” (0) and “female” (1). Childhood health ranged from 1 to 5, with higher score indicating a worse childhood health. Maternal and paternal educations ranged from 1 to 9, with a higher score indicating a better education level. Family economic status in childhood ranged from 1 to 5, with a higher score indicating a better economic level. Parent relationships ranged from 1 to 5, with a higher score indicating a better relationship. Neighborhood safety, neighborhood helping, neighborhood cleanliness were all ranged from 1 to 4, with a higher score indicating a worse neighborhood quality. Loneliness, friend playing, bullied by kids ranged from 1 to 4, with a higher score of loneliness and bullying by kids indicating a better friendship quality and with a higher score of playing with friends indicated a worse friendship quality. More details are shown in the supplementary material.

### Statistical analysis

Two stages in the statistical analysis were performed. In the first stage, a multi-trajectory modeling was used to identify the joint trajectories of 3-D health. The approach uses maximum likelihood estimation for the trajectories with discrete groups sharing different patterns^30,31^. Trajectory modeling distinguishes one outcome with several trajectories and individuals in the same group have the same trajectory. Recent improvement of multi-trajectory modeling allows for joint modeling of more than two outcomes and omission caused by mortality^29^. Each trajectory groups is now defined by several trajectories which depend on the number of outcomes, the likelihood of multi-trajectory modeling is:

$${\text{P }}\left( {{\text{Y}}_{1} ,{\text{ Y}}_{2} , \ldots ,{\text{Y}}_{n} } \right) = \mathop \sum \limits_{k} \pi_{k} \mathop \prod \limits_{n} f_{n}^{k} \left( {Y_{k} } \right)$$

where $n$ represents the number of different outcome trajectories in each trajectory group $k$. $f\left( {*} \right)$ represents the distribution for each outcome and can be different across the outcomes. Here the zero inflation poisson distributions were used for disability and depression and censor normal distribution for diseases. Model fit was identified by Odds of Correct Classification (OCC), the average posterior probability of each group based on the maximum likelihood estimates and the difference between estimated group probabilities and the actual proportion^32^. Generally, an OCC of 5 or more is recommended for all groups. The threshold of the average posterior probability is 0.7 and higher than 0.7 means an acceptable model fit. When a model fits the data well, the estimated group probabilities and the actual proportion are similar. As other similar studies, we considered the Bayesian Information Criterion (BIC) index and the explanation of each trajectory when we chose the appropriate number of potential trajectories^11,33^. The smaller the BIC value, the better the trajectory model fit is. The PROC TRAJ option was performed for SAS 9.4 to estimate the model^29–31^.

The multinomial regression model was used to analyze the association of the childhood predictors and membership in trajectory groups by using the “Glimmix” procedure for SAS 9.4. Maximum likelihood estimation was applied to the model estimation. The OR values were applied to assess the probability for older adults in different trajectory groups^15^.

## Supplementary Information

Supplementary Information
