# Supplementary material for: 3-D health trajectories and related childhood predictors among older adults in China
Source: Sci Rep. 2021 May 10;11:9874. doi: 10.1038/s41598-021-89354-6 (PMC8110566; doi:10.1038/s41598-021-89354-6)
Supplement: Supplementary file 1 — Supplementary Information [file 41598_2021_89354_MOESM1_ESM.docx]

**3-D health trajectories and related childhood predictors among older adults in China**

Chaoping Pan, PhD, Cen Wang, MD, Bhawana Shrestha, PhD, Peigang Wang*, PhD

* Correspondence author

SECTION **CESD Depression** [INTRO: The 10 items below refer to how you have felt and behaved during the last week. Every item has the same selective answers including rarely or none of the time, some, occasionally, and most or all of the time. Choose the appropriate response]

I was bothered by things that don’t usually bother me

1. Rarely or none of the time（<1 day）

2. Some or a little of the time（1-2 days）

3. Occasionally or a moderate amount of the time（3-4 days ）

4. Most or all of the time（5-7 days）

I had trouble keeping my mind on what I was doing

1. Rarely or none of the time（<1 day）

2. Some or a little of the time（1-2 days）

3. Occasionally or a moderate amount of the time（3-4 days ）

4. Most or all of the time（5-7 days）

I felt depressed

1. Rarely or none of the time（<1 day）

2. Some or a little of the time（1-2 days）

3. Occasionally or a moderate amount of the time（3-4 days ）

4. Most or all of the time（5-7 days ）

I felt everything I did was an effort

1. Rarely or none of the time（<1 day）

2. Some or a little of the time（1-2 days）

3. Occasionally or a moderate amount of the time（3-4 days ）

4. Most or all of the time（5-7 days ）

I felt hopeful about the future

1. Rarely or none of the time（<1 day）

2. Some or a little of the time（1-2 days）

3. Occasionally or a moderate amount of the time（3-4 days ）

4. Most or all of the time（5-7 days ）

I felt fearful

1. Rarely or none of the time（<1 day）

2. Some or a little of the time（1-2 days）

3. Occasionally or a moderate amount of the time（3-4 days ）

4. Most or all of the time（5-7 days ）

My sleep was restless

1. Rarely or none of the time（<1 day）

2. Some or a little of the time（1-2 days）

3. Occasionally or a moderate amount of the time（3-4 days ）

4. Most or all of the time（5-7 days ）

I was happy

1. Rarely or none of the time（<1 day）

2. Some or a little of the time（1-2 days）

3. Occasionally or a moderate amount of the time（3-4 days ）

4. Most or all of the time（5-7 days ）

I felt lonely

1. Rarely or none of the time（<1 day）

2. Some or a little of the time（1-2 days）

3. Occasionally or a moderate amount of the time（3-4 days ）

4. Most or all of the time（5-7 days ）

I could not get “going”

1. Rarely or none of the time（<1 day）

2. Some or a little of the time（1-2 days）

3. Occasionally or a moderate amount of the time（3-4 days ）

4. Most or all of the time（5-7 days ）

**Disease**

Have you been diagnosed with Hypertension by a doctor?

1. Yes

2. No

Have you been diagnosed with Dyslipidemia (elevation of low density lipoprotein, triglycerides (TGs),and total cholesterol, or a low high density lipoprotein level) by

a doctor?

1. Yes

2. No

Have you been diagnosed with Diabetes or high blood sugar by a doctor?

1. Yes

2. No

Have you been diagnosed with Cancer or malignant tumor (excluding minor skin cancers) by a doctor?

1. Yes

2. No

Have you been diagnosed with Chronic lung diseases, such as chronic bronchitis, emphysema (excluding tumors, or cancer) by a doctor?

1. Yes

2. No

Have you been diagnosed with Liver disease (except fatty liver, tumors, and cancer) by a doctor?

1. Yes

2. No

Have you been diagnosed with Heart attack, coronary heart disease, angina, congestive heart failure, or other heart problems by a doctor?

1. Yes

2. No

Have you been diagnosed with Stroke by a doctor?

1. Yes

2. No

Have you been diagnosed with Kidney disease (except for tumor or cancer) by a doctor?

1. Yes

2. No

Have you been diagnosed with Stomach or other digestive diseases (except for tumor or cancer) by a doctor?

1. Yes

2. No

Have you been diagnosed with Emotional, nervous, or psychiatric problems by a doctor?

1. Yes

2. No

Have you been diagnosed with Arthritis or rheumatism by a doctor?

1. Yes

2. No

Have you been diagnosed with Asthma by a doctor?

1. Yes

2. No

**Disability**

The **Instrumental Activities of Daily Living (IADLs)** included are managing money, taking medications, shopping for groceries, preparing meals, cleaning house. A code of 0 indicates that the respondent did not report any problems with the instrumental activity or that the respondent was not asked the question because they previously reported no difficulty jogging, getting up from chair, climbing, stooping, reaching, lifting/carrying, and picking up a coin. A code of 1 indicates that the respondent reported some difficulty with the activity or could not do the activity.

The **Activities of Daily Living (ADLs)** included are dressing, bathing and showering, eating, getting in and out of bed, using the toilet and controlling urination and defecation. A code of 0 indicates that the respondent did not report any problems with the activity. A code of 1 indicates that the respondent reported some difficulty with the activity or could not do the activity.

**Demographic factors**

The respondent’s gender is set to 0 for male and 1 for female.

Before you were 15 years old (including 15 years old), would you say that compared

to other children of the same age, you were

1. Much healthier

2. Somewhat healthier

3. About average

4. Somewhat less healthy

5. Much less healthy

**Family status**

What is the highest level of education your biological mother completed?

1. No formal education (illiterate)

2. Did not finish primary school but capable of reading or writing

3. Sishu/home school

4. Graduate from elementary school

5. Graduate from middle school

6. Graduate from high school

7. Graduate from vocational school

8. Graduate from Two/Three Year College / Associate degree

9. Graduate from Four Year College / Bachelor’s degree

What is the highest level of education your biological father completed?

1. No formal education (illiterate)

2. Did not finish primary school but capable of reading or writing

3. Sishu/home school

4. Graduate from elementary school

5. Graduate from middle school

6. Graduate from high school

7. Graduate from vocational school

8. Graduate from Two/Three Year College / Associate degree

9. Graduate from Four Year College / Bachelor’s degree

How would you rate the relationship your parents had with each other when you were

growing up? Is it excellent, very good, good, fair or poor?

1. Excellent

2. Very good

3. Good

4. Fair

5. Poor

When you were a child before age 17, compared to the average family in the same

community/village at that time, how was your family’s financial situation?

[If your family lived separately and had different financial situations, answer for the

family you lived with the longest time.]

1. A lot better off than them

2. Somewhat better off than them

3. Same as them

4. Somewhat worse off than them

5. A lot worse off than them

**Childhood Neighborhood Quality**

Was it safe being out alone at night in the neighborhood where you lived as a child? Is it very safe, somewhat safe, not very safe or not safe at all?

1. Very safe

2. Somewhat safe

3. Not very safe

4. Not safe at all

Were the neighbors of the place where you lived as a child willing to help each other out? Is it very willing to, somewhat willing to, not very willing to or not willing to?

1. Very willing to

2. Somewhat willing to

3. Not very willing to

4. Not willing to at all

Was the neighborhood of the place where you lived as a child very clean and attractive? Is it very clean and attractive, somewhat clean and attractive, not very clean and attractive or not clean and attractive at all?

1. Very clean and attractive

2. Somewhat clean and attractive

3. Not very clean and attractive

4. Not clean and attractive at all

**Childhood Friendship and Experience**

When you were a child, how often did you feel lonely for not having friends? Is it often, sometimes, not very often or never?

1. Often

2. Sometimes

3. Not very often

4. Never

When you were a child, did you often have a group of friends that you felt comfortable spending time with? Is it often, sometimes, not very often or never?

1. Often

2. Sometimes

3. Not very often

4. Never

When you were a child, how often were you picked on or bullied by kids in your neighborhood? Is it often, sometimes, rarely or never?

1. Often

2. Sometimes

3. Not very often

4. Never

**Adults variables**

**Respondent’s age** is calculated by the respondent’s birth year and month minus the interview year and month. In wave 1, self-reported age is used if there is a missing value for the calculated age.

**the highest level of education** that the respondent has attained is defined using the following codes: 1.No Formal Education (Illiterate), 2.Did Not Finish Primary School but can Read, 3.Sishu (Private Tutoring), 4.Elementary School, 5.Middle School, 6.High School, 7.Vocational School, 8.Two/three-year college, 9.College Grad and 10.Post-graduate degree. Don’t know, missing, or refused values are assigned special missing codes (.d), (.m), (.r), respectively.

**Residence** is based on the information recorded in PSU file at the community level; that is, whether the region is rural or urban is defined by National Bureau of Statistics of the People's Republic of China. A code of 0 indicates the household is located in a rural region and a code of 1 indicates the household is located in an urban region.

As for **marital status**, respondents are asked to indicate their marital status. Categories include: (1) Married with spouse present, (2) Married but not living with spouse temporarily for reasons such as work, (3) Separated, (4) Divorced, (5) Widowed, or (6) Never married. A code of 0 indicates that the respondent reports had no spouse. A code of 1 indicates that the respondent reports had a spouse.

**Smoking** was measured by how many cigarettes the respondent smokes on average per day. all respondents who report ever having smoked are asked what products they did/do normally smoke and given the options of smoking a pipe, smoking self-rolled cigarettes, filtered cigarettes, unfiltered cigarettes, cigars, and water cigarettes. Respondents are then asked whether they still smoke or they have quit. All respondents who report normally using filtered cigarettes or unfiltered cigarettes and report still smoking are asked in one day about how many cigarettes they consume. A code of 0 indicates that the respondent reports never smoking. A code of 1 indicates that the respondent reports smoking at some point.

**Drinking** means the highest frequency of drinking behavior during the last year that the respondent reports for any one of the three types of alcohol. Drinking is defined as 0. none or doesn't drink, 1. Once a month, 2. 2 to 3 days a month, 3. Once a week, 4. 2 to 3 days a week, 5.4 to 6 days a week, 6.Daily, 7.Twice a day and 8.More than twice a day. Don’t know, or refused values of drinking is assigned special missing values (.d), (.r), respectively. Drinking is set to plain missing (.) for respondents who did not respond to this wave. A code of 0 indicates that the respondent reports never having an alcoholic drink in the past. A code of 1 indicates that the respondent reports having an alcoholic drink in the past.

**Medical insurance** indicates whether the respondent is covered by any public health insurance program. Below is the list of the health insurance plans:

1. Urban employee medical insurance (yi-bao)

2. Urban resident medical insurance

3. New cooperative medical insurance(he-zuo-yi-liao)

4. Urban and rural resident medical insurance

5. Government medical insurance(gong-fei)

6. Medical aid

7. Urban non-employed persons's health insurance

A code of 0 indicates that the respondent is not covered by any public health insurance plan. A code of 1 indicates that the respondent is covered by at least one type of public health insurance plan.

**Pension** indicates whether the respondent is currently receiving a public pension without disability. In Wave 1, public pension is comprised of pension benefit from social insurance agency, rural pension, residents’ pension, urban residents’ pension, new rural social pension insurance, pension subsidy to the oldest old, and new rural social pension insurance. A code of 0 indicates that the respondent reports did not have a pension. A code of 1 indicates that the respondent reports have pension.
